# Comprehensive analysis of the differences between left- and right-side colorectal cancer and respective prognostic prediction

**DOI:** 10.1186/s12876-022-02585-3

**Published:** 2022-11-23

**Authors:** Mengye Niu, Chengyang Chen, Xian Gao, Yi Guo, Bingzhou Zhang, Xin Wang, Shihao Chen, Xupeng Niu, Chao Zhang, Like Li, Zhongxin Li, Zengren Zhao, Xia Jiang

**Affiliations:** 1 Department of General Surgery, The First Hospital of Hebei Medical University, No. 89 Donggang Street, Yuhua District, Shijiazhuang, Hebei China; 2 Hebei Key Laboratory of Colorectal Cancer Precision, The First Hospital of Hebei Medical University, Shijiazhuang, China; 3 Department of Pathology, The First Hospital of Hbei Medical University, Shijiazhuang, China

**Keywords:** Left-sided colon cancer, Right-sided colon cancer, Biomarkers, Nomogram, Immune microenvironment, Tumor mutation burden, Immune checkpoint

## Abstract

**Background:**

Previous studies have reported that the tumor heterogeneity and complex oncogenic mechanisms of proximal and distal colon cancer (CRC) are divergent. Therefore, we aim to analyze the differences between left-sided CRC (L_cancer) and right-sided CRC (R_cancer), as well as constructing respective nomograms.

**Methods:**

We enrolled 335 colon cancer patients (146 L_cancer patients and 189 R_cancer patients) from The Cancer Genome Atlas (TCGA) data sets, and 102 pairs of color cancer tissue and adjacent normal tissue (51 L_cancer patients and 51 R_cancer patients) from our hospital. Firstly, we analyzed the differences between the L_cancer patients and R_cancer patients, and then established the L_cancer and R_cancer prognostic models using LASSO Cox.

**Results:**

R_cancer patients had lower survival than L_cancer patients. R_cancer patients had higher ESTIMATE and immune scores and lower tumor purity. These patterns of expression of immune checkpoint-related genes and TMB level were higher in R_cancer than in L_cancer patients. Finally, we using Lasso Cox regression analyses established a prognostic model for L_cancer patients and a prognostic model for R_cancer patients. The AUC values of the risk score for OS in L_cancer were 0.862 in the training set and 0.914 in the testing set, while those in R_cancer were 0.835 in the training set and 0.857 in the testing set. The AUC values in fivefold cross-validation were between 0.727 and 0.978, proving that the two prognostic models have great stability. The nomogram of L_cancer included prognostic genes, age, pathological M, pathological stage, and gender, the AUC values of which were 0.800 in the training set and 0.905 in the testing set. Meanwhile, the nomogram of R_cancer comprised prognostic genes, pathological N, pathological T, and age, the AUC values of which were 0.836 in the training set and 0.850 in the testing set. In the R_cancer patients, high-risk patients had a lower proportion of ‘B cells memory’, ‘Dendritic cells resting’, immune score, ESTIMATE score, immune checkpoint-related genes, and HLA-family genes, and a higher proportion of ‘T cells follicular helper’, ‘Dendritic cells activated’, and ‘Mast cells activated’.

**Conclusions:**

We found significant differences between L_cancer and R_cancer patients and established a clinical predictive nomogram for L_cancer patients and a nomogram for R_cancer patients. Additionally, R_cancer patients in low-risk groups may be more beneficial from immunotherapy.

**Supplementary Information:**

The online version contains supplementary material available at 10.1186/s12876-022-02585-3.

## Introduction

Colon cancer (CRC) is one of the most common cancers and cause of cancer death globally, seriously endangering the health of patients [1]. In recent years, there has been a growing body of evidence demonstrating that the primary tumor location of CRC is an important prognostic factor, owing to distinct biological features [2–4]. Despite the fact that the primary tumor site is not generally considered in CRC management, left-sided colon cancers (L_cancer) and right-sided colon cancers (R_cancer) exhibit different clinical and biological characteristics [5]. A meta-analysis of 66 studies with more than 1.4 million patients with a median follow-up of 65 months revealed that the tumor side had a significant prognostic impact on overall survival, with a 20% percent longer life expectancy, independent of stage, race, adjuvant chemotherapy, year of study, number of participants, and quality of included studies. [6]. The differences in colon cancer by its location have been identified through extensive research, including survival, tumor microenvironment, methylation profile, microbiota, gene expression, and epigenetic changes. [2, 3, 6–8]. In addition, the tumor location also influences the outcome of adjuvant chemotherapy, palliative therapy, or targeted therapy. Therefore, it is of special significance to classify CRC by its location.

Nomograms are widely used for prognosis in CRC patients. However, few previous studies have separately built predictive models to predict patient prognosis with respect to location. In this study, we separately build predictive models for L_cancer and R_cancer, identifying potential prognostic biomarkers for left and right CRC. Age, sex, histological classification, and so forth, are also important factors that can influence clinical outcomes and can improve the accuracy of models. Therefore, we also aimed to analyze the differences between L_cancer and R_cancer and construct respective nomograms for L_cancer and R_cancer, containing prognostic gene signatures and clinical prognostic factors, which are expected to allow for more accurate predictions in the prognosis of CRC, facilitating accurate diagnosis and treatment.

## Material and methods

### Data sets

The transcriptome data, somatic mutation data, and clinical information of CRC patients were downloaded from The Cancer Genome Atlas (TCGA, https://portal.gdc.cancer.gov/), which includes transcriptome data for 332 CRC patients (146 L_cancer patients and 189 R_cancer patients) and somatic mutation data for 329 CRC patients (142 L_cancer patients and 187 R_cancer patients).

L_cancer patients were divided into L_cancer training and L_cancer internal validation sets at a ratio of 7:3. The L_cancer external validation set contained those who operated in our hospital, including 51 L_cancer patients.

R_cancer patients were also divided into R_cancer training set and R_cancer internal validation sets at a ratio of 7:3. The R_cancer external validation set contained those who operated in our hospital, including 51 R_cancer patients.

A total of 102 pairs of colon cancer and adjacent normal control samples were stored at − 80 °C. Patients were followed up by telephone interviews. As of the final data cutoff, December 30, 2021, the median duration of follow-up in the study was 4.5 years and the criterion to proceed with the final OS analysis was met.

The term "R_cancer" refers to any (histologically confirmed) adenocarcinoma arising from the caecum, ascending colon, or hepatic flexure. Any tumor that arises in the splenic flexure, descending colon or sigmoid colon was referred to as L_cancer.

### Survival analysis

Using Kaplan–Meier survival analysis, we evaluated the differences in survival between patients with different clinicopathological characteristics, between high-risk and low-risk groups and between the L_cancer and R_cancer groups in the data sets mentioned above. The ‘survival’ package in R was used to perform a two‐sided log‐rank test and univariate and multivariate Cox regression analyses [9].

### Differential gene analysis and functional annotation

By using the "edgeR" package in R, we identified differentially expressed genes (DEGs) between L_cancer and R_cancer, L_cancer and L_normal, R_cancer and R_normal based on differential expression analysis. To screen for DEGs, |log2 FC (fold-change)|> 1 and *P* < 0.05 were set as thresholds. To investigate the possible biological processes, cellular components, and molecular functions of DEGs, GO enrichment and KEGG pathway analyses were performed by using the R software package “clusterProfiler” [10–12].

### Gene set variation analysis (GSVA)

By using the "GSVA" package in R, we evaluated the t-scores and assigned pathway activity conditions to L_cancer and R_cancer patients to reveal pathway enrichment. The "limma" package in R was also used to show differences in pathway activation between L_cancer and R_cancer patients [13–15].

### The proportion of immune cell infiltration and the calculation of tumor purity

In each cancer sample, the relative proportions of 22 immune cell types were calculated using the CIBERSORT software [16]. A file called "LM22.txt", containing 547 gene signatures (https://cibersort.stanford.edu/download.php), is also needed in R. ESTIMATE was used to calculate immune, stromal, and ESTIMATE scores, as well as tumor purity, based on Yoshihara et al. [17].

### Profiles of tumor mutation burden (TMB) and correlation analysis

The TMB was defined as: TMB = (total count of variants)/(the whole length of exons). In a waterfall plot, the mutation profiles of two groups were compared using the maftools package [18]. Afterward, the difference in mutation frequencies between the two groups was measured with the chi-square test. TMB was derived for each patient, calculated using Pearson correlation analysis with estimated *P*-values.

### LASSO cox regression analysis

LASSO Cox regression analysis with the R package glmnet was then used to identify hub genes associated with the prognosis of L_cancer or R_cancer, and a Risk Score was calculated for each sample using the screened hub genes following the following formula [19]:

$$Riskscore = \sum\limits_{i = 1}^{N} {\left( {Expi \times Coef} \right)}$$

where N represents the number of signature genes, Expi is the gene expression levels, and Coef is the estimated regression coefficient value from the Cox proportional-hazards analysis. Based on this optimal cutoff value, the R survival package "survminer" was used to divide patient groups into Low- and High-Risk groups. Moreover, model predictive power was evaluated by calculating the AUC of 1-, 3-, 5-, 7-year, and all time-dependent ROC curves, using the “survivalROC” package.

### Building and validating a predictive nomogram

To construct the nomograms, we used univariate and multivariate Cox regression analyses. Forest plots were used to display the *P*-value, HR, and 95% CI for each variable, using R's 'forest plot' package. Based on independent prognostic factors, the nomograms were generated in R using the rms, nomogramEx, and ggDCA packages. In the next step, Using calibration curves, we determined whether the predicted survival outcome matched the actual outcome. Moreover, training set decision curve analysis (DCA) and internal validation set DCA, which is a statistical method for assessing and comparing predictive models, was used to determine the clinical suitability of our established nomograms.

### RNA isolation and quantitative reverse transcription PCR assay

For total RNA isolation, the TRIzol reagent by Invitrogen was used, and for complementary DNA synthesis, the PrimeScript RT reagent kit by Takara was used. RT-PCR was carried out using SYBR Premix Ex Taq I. GAPDH served as an internal control. Relative RNA abundances were calculated by using the standard 2-ΔCt method.

### Statistical analysis

A two-sided significance level of 0.05 was used to determine statistical significance in all analyses using R software (version 3.6.3). All significance levels were two-sided.

## Results

### Differences between L_cancer and R_cancer patients

#### Differences in demographic characteristics between L_cancer and R_cancer patients

**Fig. 1:**
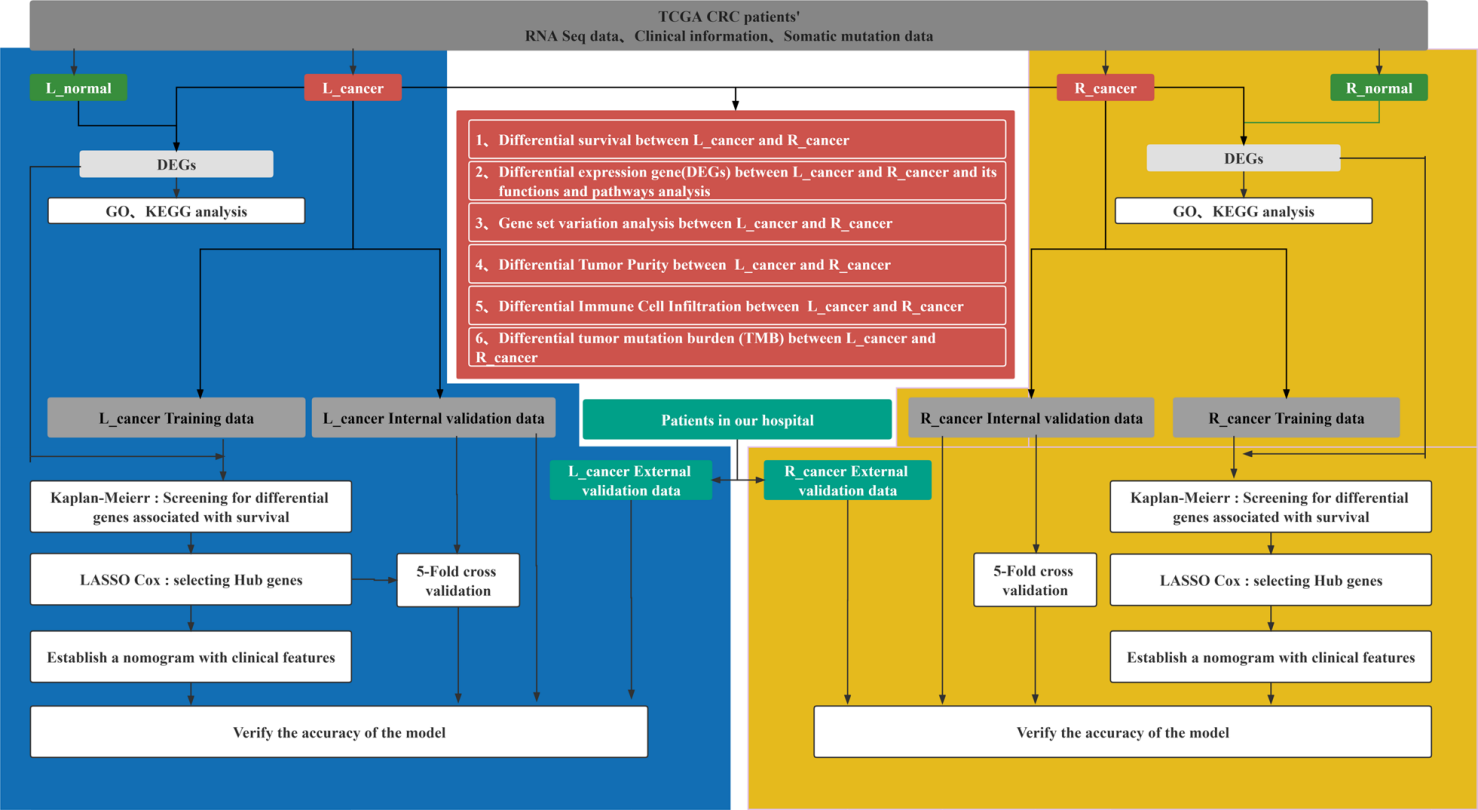
The flow diagram shows that: 1 the difference between L_cancer and R_cancer; 2 Nomograms were established to predict the prognosis of L_cancer and R_cancer, respectively. (L_cancer, left-side colon cancer; R_cancer, right-side colon cancer)

**Fig. 2:**
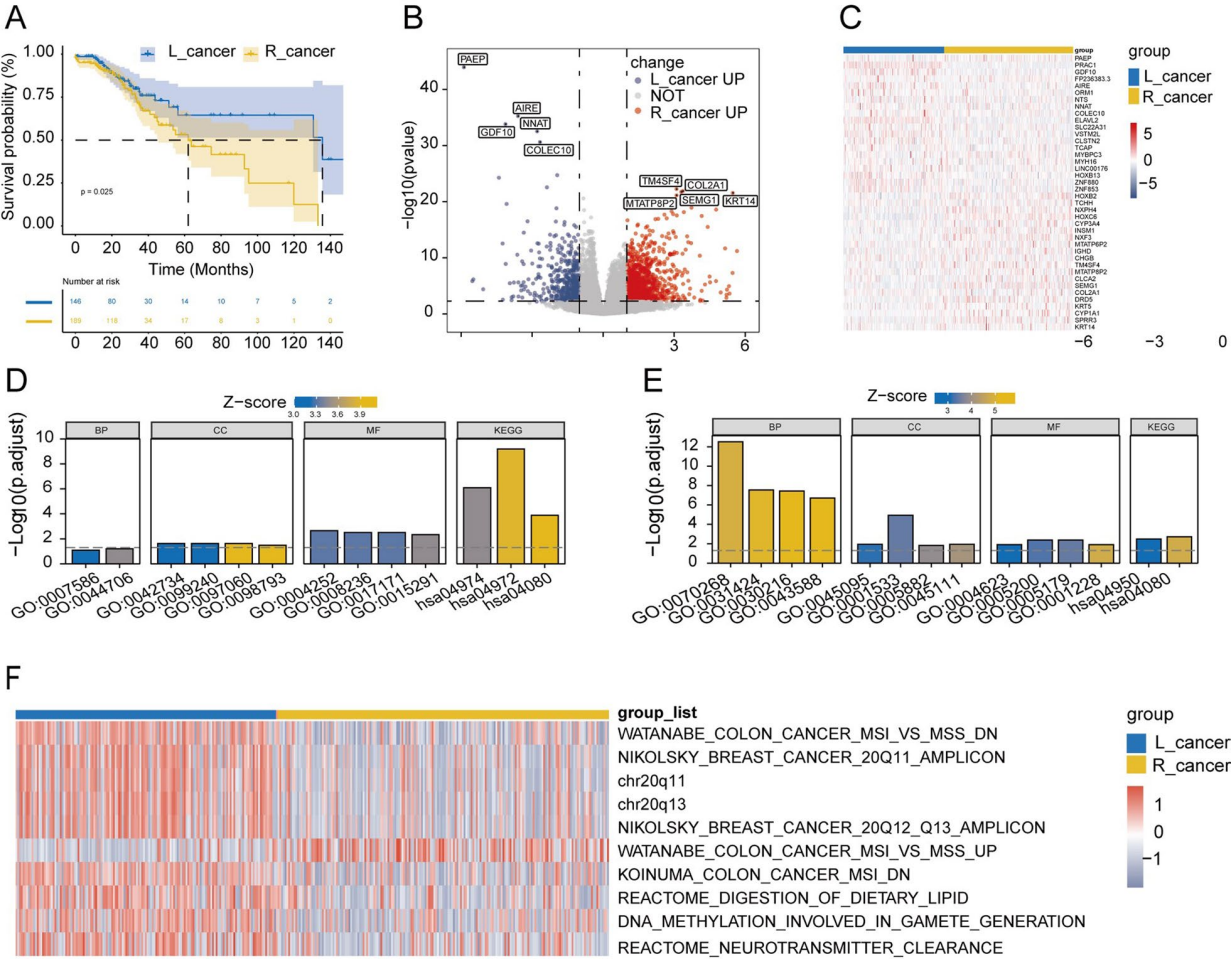
Differentially expressed genes and functional annotation between L_cancer and R_cancer patients. **A** Survival rates difference between L_cancer patients and R_cancer patients. **B** Volcano plot for differentially expressed genes (DEGs) of L_cancer patients and R_cancer patients. **C** Heatmap plot for top 40 DEGs of the two groups. **D**, **E** GO enrichment analysis and KEGG analysis of the up-regulated DEGs in **D** L_cancer and **E** R_cancer. **F** Heatmap demonstrated the top 10 different gene set enrichment analysis (GSVA) pathways of the two groups

An overview of the steps is presented as a flow chart in Fig. 1. The demographic characteristics of patients are summarized in Table 1. The L_cancer patients found a significant difference between R_cancer patients regarding age, stage N, and survival rate (*P* < 0.05). It is noteworthy that we observed lower survival after R_cancer versus L_cancer (Fig. 2A).

**Table 1:** Demographic and clinical characteristics of patients

	L_cancer			*P* Value	R_cancer			*P* Value	TCGA data sets and Patients in our hospital		*P* Value
	TCGA data sets		Patients in our hospital		TCGA data sets		Patients in our hospital				
	Training set	Internal validation set	External validation set		Training set	Internal validation set	External validation set		Total L_cancer	Total R_cancer	
	(n = 103)	(n = 43)	(n = 51)		(n = 133)	(n = 56)	(n = 51)		(n = 197)	(n = 240)	
*Age (y)*				0.064				0.067			**0.014**
< 65	40 (38.8%)	20 (46.5%)	30 (58.8%)		37 (27.8%)	23 (41.1%)	22 (43.1%)		90 (45.7%)	82 (34.2%)	
≥ 65	63 (61.2%)	23 (53.5%)	21 (41.2%)		96 (72.2%)	33 (58.9%)	29 (56.9%)		107 (54.3%)	158 (65.8%)	
*Gender*				0.679				0.532			0.198
Female	52 (50.5%)	24 (55.8%)	24 (47.1%)		59 (44.4%)	28 (50.0%)	20 (39.2%)		100 (50.8%)	107 (44.6%)	
Male	51 (49.5%)	19 (44.2%)	27 (52.9%)		74 (55.6%)	28 (50.0%)	31 (60.8%)		97 (49.2%)	133 (55.4%)	
*T*				< 0.001				< 0.001			0.127
T1	2 (1.9%)	3 (7.0%)	4 (7.8%)		3 (2.26%)	1 (1.8%)	0 (0.00%)		9 (4.6%)	4 (1.7%)	
T2	13 (12.6%)	11 (25.6%)	1 (2.0%)		22 (16.5%)	11 (19.6%)	2 (3.9%)		25 (12.7)	35 (14.6%)	
T3	81 (78.6%)	26 (60.5%)	16 (31.4%)		88 (66.2%)	38 (67.9%)	11 (21.6%)		123 (62.4%)	137 (57.1%)	
T4	7 (6.8%)	3 (7.0%)	30 (58.8%)		20 (15.0%)	6 (10.7%)	38 (74.5%)		40 (20.3%)	64 (26.7%)	
*N*				0.992				0.169			**0.018**
N0	56 (54.4%)	22 (51.2%)	26 (51.0%)		84 (63.2%)	34 (60.7%)	23 (45.1%)		104 (52.8%)	141 (58.8%)	
N1	31 (30.1%)	14 (32.6%)	16 (31.4%)		25 (18.8%)	8 (14.3%)	14 (27.5%)		61 (31.0%)	47 (19.6%)	
N2	16 (15.5%)	7 (16.3%)	9 (17.6%)		24 (18.0%)	14 (25.0%)	14 (27.5%)		32 (16.2%)	52 (21.7%)	
*M*				0.946				0.590			0.632
M0	87 (84.5%)	36 (83.7%)	42 (82.4%)		115 (86.5%)	49 (87.5%)	41 (80.4%)		165 (83.8%)	200 (83.3%)	
M1	16 (15.5%)	7 (16.3%)	9 (17.6%)		18 (13.5%)	7 (12.5%)	10 (19.6%)		32 (16.2%)	40 (16.7%)	
*Stage*				0.007				0.166			0.834
Stage I	10 (9.7%)	13 (30.2%)	3 (5.9%)		22 (16.5%)	12 (21.4%)	2 (3.9%)		26 (13.2%)	36 (15.0%)	
Stage II	43 (41.7%)	8 (18.6%)	22 (43.1%)		56 (42.1%)	20 (35.7%)	19 (37.3%)		73 (37.1%)	95 (39.6%)	
Stage III	34 (33.0%)	15 (34.9%)	17 (33.3%)		37 (27.8%)	17 (30.4%)	20 (39.2%)		66 (33.5%)	74 (30.8%)	
Stage IV	16 (15.5%)	7 (16.3%)	9 (17.6%)		18 (13.5%)	7 (12.5%)	10 (19.6%)		32 (16.2%)	35 (14.6%)	
*Survival*				0.991				0.686			**0.047**
Alive	84 (81.6%)	35 (81.4%)	42 (82.4%)		96 (72.2%)	41 (73.2%)	40 (78.4%)		161 (81.7%)	177 (73.8%)	
Dead	19 (18.4%)	8 (18.6%)	9 (17.6%)		37 (27.8%)	15 (26.8%)	11 (21.6%)		36 (18.3%)	63 (26.3%)	

Statistically significant values are shown in bold

Dates were displayed in counts (%); L_cancer: Left-side colon cancer; R_cancer: Right-side colon cancer; TCGA: The Cancer Genome Atlas

Moreover, there is no difference between the training set and the verification set except T stage. The difference in the T stage may due to the poor stage of patients from our hospital, but it does not affect the internal validation.

#### Differential expressed genes and functional annotation between L_cancer and R_cancer patients

By comparing the transcriptome data, we identified 540 significantly up-regulated DEGs in the L_cancer group and 1507 significantly up-regulated DEGs in the R_cancer group (Fig. 2B). The heatmap was shown the top 40 DEGs with the greatest variation (Fig. 2C).

Further, we applied the DEGs for functional enrichment analysis. L_cancer up-regulated DEGs were enriched in 38 GO terms and 3 KEGG pathways (FDR < 0.5, Fig. 2D), while R_cancer up-regulated DEGs were enriched in 129 GO terms and 2 KEGG pathways (FDR < 0.5, Fig. 2E).

In addition, GSVA revealed that MIS vs. MSS, 20Q11 anplicon chr20q11, chr20q13, reactome digestion of dietary lipids, DNA methylation involved in gamete generation and so on were different in L_cancer and R_cancer patients (|log_2_FC|> 0.2, all *P* < 0.05; Fig. 2F).

#### Differential immune microenvironment between L_cancer and R_cancer patients

By comparing the immune microenvironments between L_cancer and R_cancer patients, significant differences were observed between the two groups with regard to immune infiltration components.

**Fig. 3:**
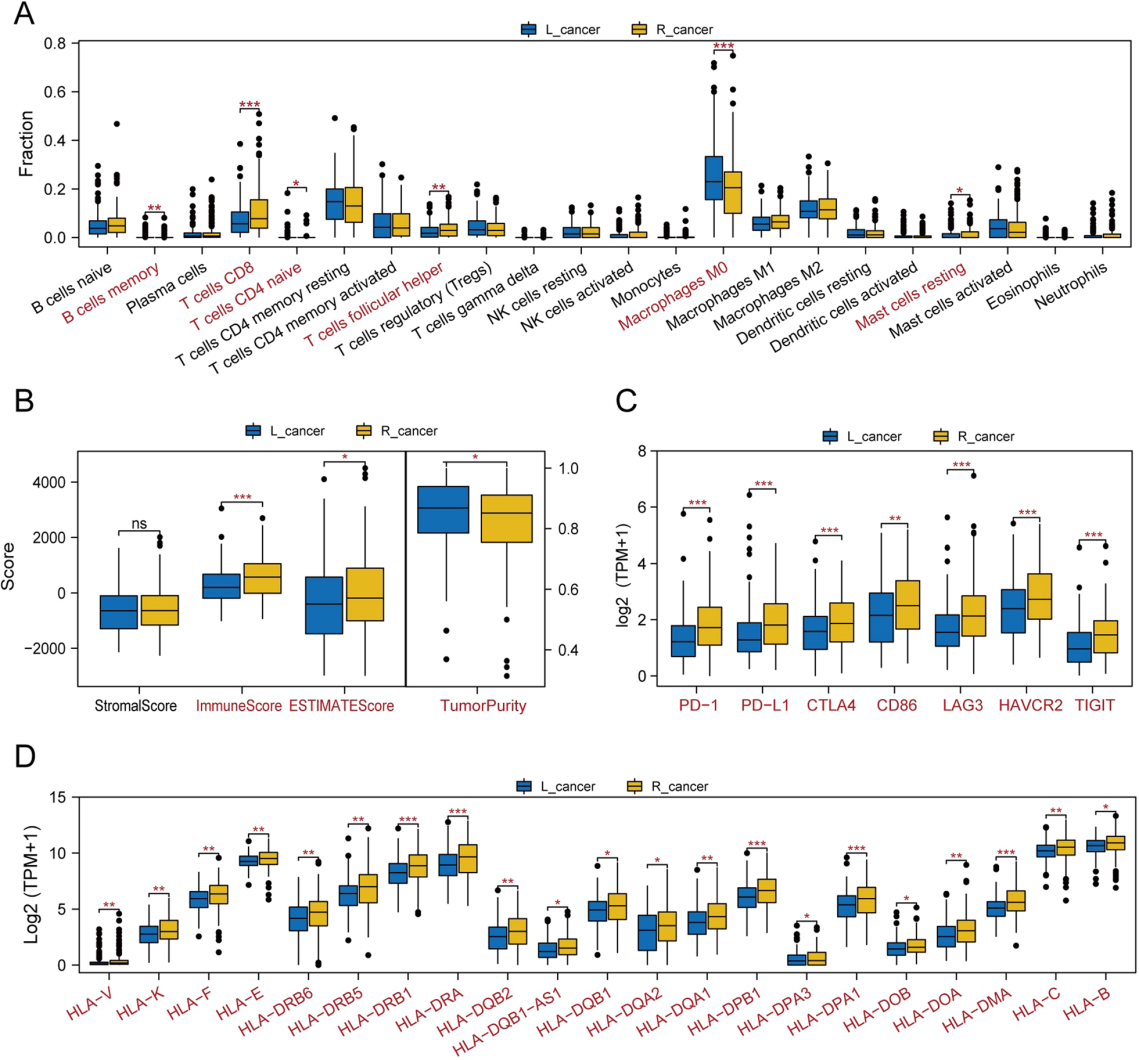
Differential immune microenvironment between L_cancer and R_cancer patients. **A** The comparison of immune infiltration levels between L_cancer and R_cancer patients, based on CIBERSORT. **B** The Stromal Score difference, Immune Score difference, ESTIMATE Score difference, and tumor purity difference between L_cancer and R_cancer patients. **C** The immune checkpoint-related gene expression levels in L_cancer and R_cancer patients. **D** HLA-related gene expression level in L_cancer and R_cancer patients. *Notes*: ns *P* > 0.05, *P < 0.05, **P < 0.01, ***P < 0.001

In the R_cancer patients, the proportions of ‘T cell CD8’, ‘T cells CD4 naïve’, ‘T cells follicular helper’, ‘Mast cells resting’ were significantly higher and ‘B cells memory’, ‘macrophages M0’ were lower than in L_cancer patients (Wilcoxon test, all *P* < 0.05; Fig. 3A).

Comparing the Stromal score, ESTIMATE score, immune score, and tumor purity of L_cancer and R_cancer patients, we found that the R_cancer patients had a lower tumor purity and higher ESTIMATE and immune scores (Wilcoxon test, *P* < 0.05; Fig. 3B) than L_cancer patients.

We also analyzed the immune checkpoint-related genes (PD-1, PD-L1, CTLA4, CD86, LAG3, HAVCR2, TIGIT) and HLA family-related genes levels, which are considered biomarkers for predicting the efficacy of immunotherapy, between L_cancer and R_cancer patients and found that the expression levels of immune checkpoint-related genes and HLA family-related genes were significantly higher in R_cancer patients (Wilcoxon test, all *P* < 0.05; Fig. 3C, D).

#### Differential TMB landscape between L_cancer and R_cancer

**Fig. 4:**
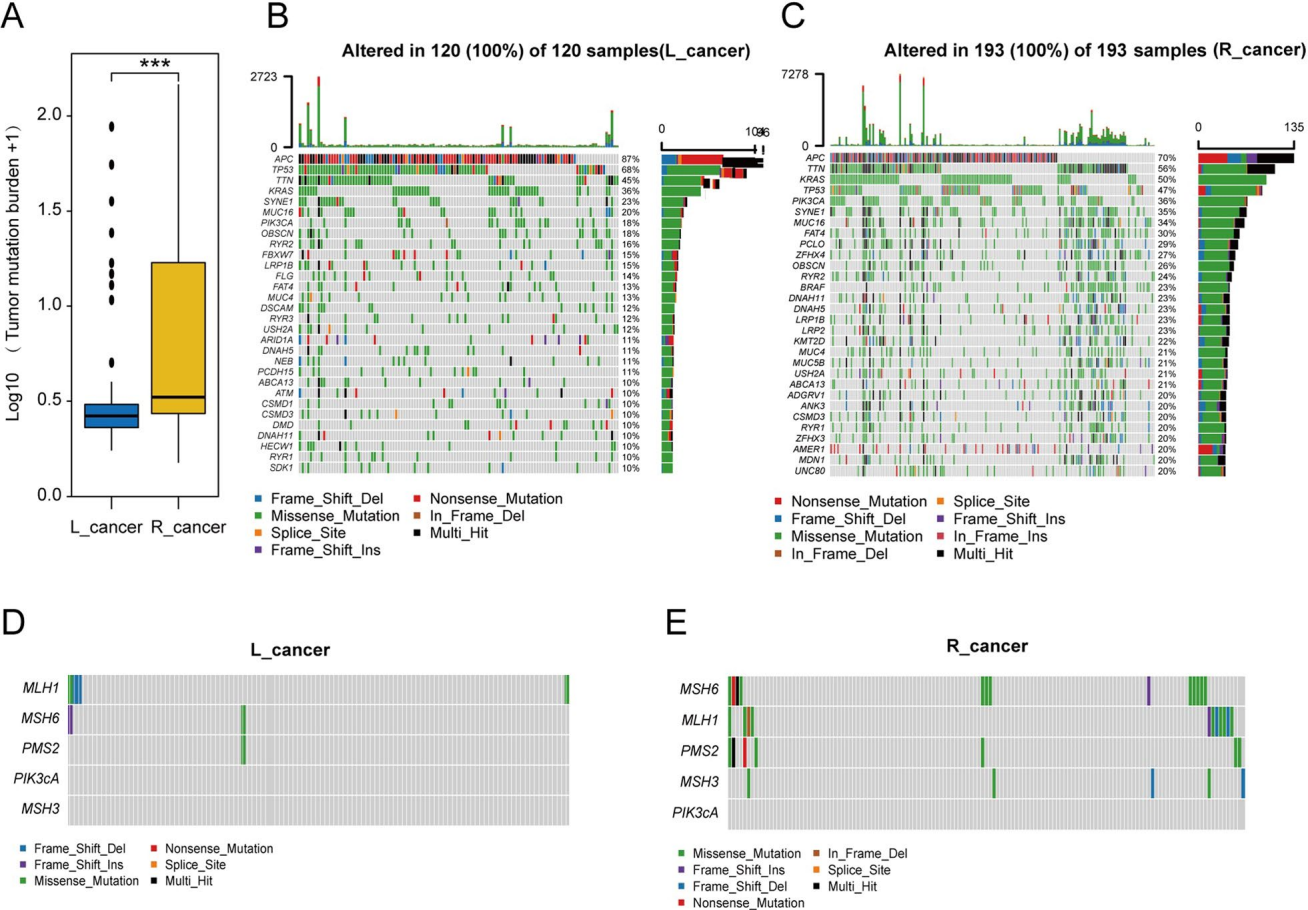
Differential TMB landscape between L_cancer and R_cancer patients. **A** The tumor mutation burden difference between L_cancer and R_cancer patients. **B**, **C** waterfall lot demonstrated the top 30 frequently mutated genes in **B** L_cancer and **C** R_cancer patients. **D**, **E** The mutation of microsatellite instability(MSI)-related genes in L_cancer and R_cancer patients. *Notes*: ***P < 0.001

The mutation prevalence varied dramatically within CRC in different locations. The mutation frequency in R_cancer patients was relatively higher than that in L_cancer patients (Fig. 4A). Moreover, the L_cancer and R_cancer groups contained different mutant genes. Waterfall plots (Fig. 4B, C) show the first 30 gene mutation rates in each location. A major discrepancy can be seen, as TP53 presented a higher mutation rate in L_cancer (L_cancer, 68%; R_cancer, 48%), while PIK3CA (L_cancer, 18%; R_cancer, 33%) and KRAS (L_cancer, 36%; R_cancer, 46%) showed higher yield mutation rates in R_cancer.

We analyzed microsatellite instability (MSI)-related genes’ mutation in each group, which showed that the L_cancer patient had MSI (Fig. 4D, E).

### Identifying DEGs and functional annotation in tumor and normal patients

**Fig. 5:**
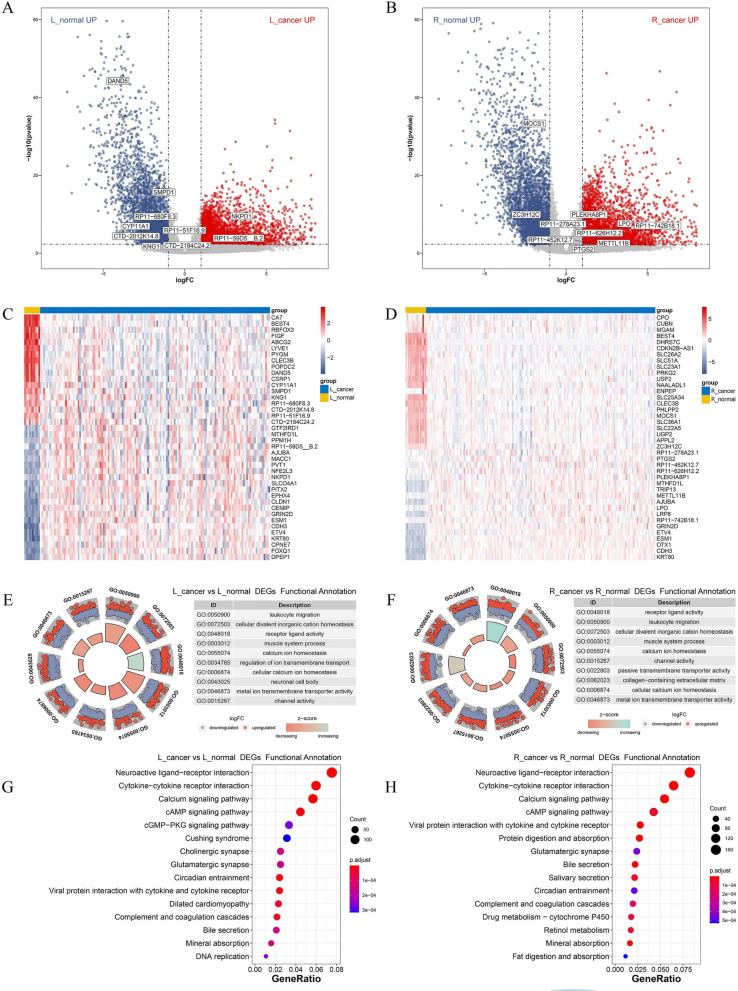
Identifying DEGs and Functional Annotation in Tumor and Normal Patients. **A** Volcano plot for DEGs between L_cancer and L_normal patients. **B** Volcano plot for DEGs between R_cancer and R_narmal patients. **C** Heatmap of the top 40 DEGs between L_cancer and L_normal patients. **D** Heatmap of the top 40 DEGs between R_cancer and R_narmal patients. **E** GO enrichment analysis of the DEG between L_cancer and L_normal patients. **F** GO enrichment analysis of the DEG between R_cancer and R_narmal patients. **G** Top 15 KEGG analysis of the DEG between L_cancer and L_normal patients. **H** Top 15 KEGG analysis of the DEG between R_cancer and R_narmal patients

By comparing the transcriptome data of L_cancer and L_normal groups, we identified 4788 up-regulated DEGs and 4062 down-regulated DEGs (Fig. 5A). The top 20 up-regulated and down-regulated genes were displayed by heatmap (Fig. 5C). Further, we analyzed these DEGs between L_cancer and L_normal groups for functional enrichment analysis. This evaluation revealed the enrichment of 1139 GO terms and 65 KEGG pathways (FDR < 0.05). We chose to show the top 10 GO terms and 15 KEGG pathways in Fig. 5E, G.

Likewise, the DEGs between R_cancer and R_normal identified 6261 up-regulated DEGs and 4501 down-regulated DEGs (Fig. 5B). The top 20 up-regulated and down-regulated genes were displayed by heatmap (Fig. 5D). These DEGs between R_cancer and R_normal groups be analyzed for functional enrichment analysis. A total of 1072 GO terms and 61 KEGG pathways had been enriched (FDR < 0.05). We chose to show the top 10 GO terms and 15 KEGG pathways in Fig. 5F, H.

### Construction of prognostic gene model

To identify prognosis-related genes, we first screened genes using the Kaplan–Meier method in DEGs with *P* < 0.05, in order to screen survival-related DEGs as candidate genes affecting prognosis. Then, to avoid model overfitting, we performed a multivariate Cox regression analysis with the LASSO penalty algorithm to solve the multi-collinearity problem. Finally, we obtained 10 genes associated with the prognosis of L_cancer patients and 10 genes associated with the prognosis of R_cancer patients. These genes have a significant impact on the survival of patients (Additional file 1: Fig. S1).

**Fig. 6:**
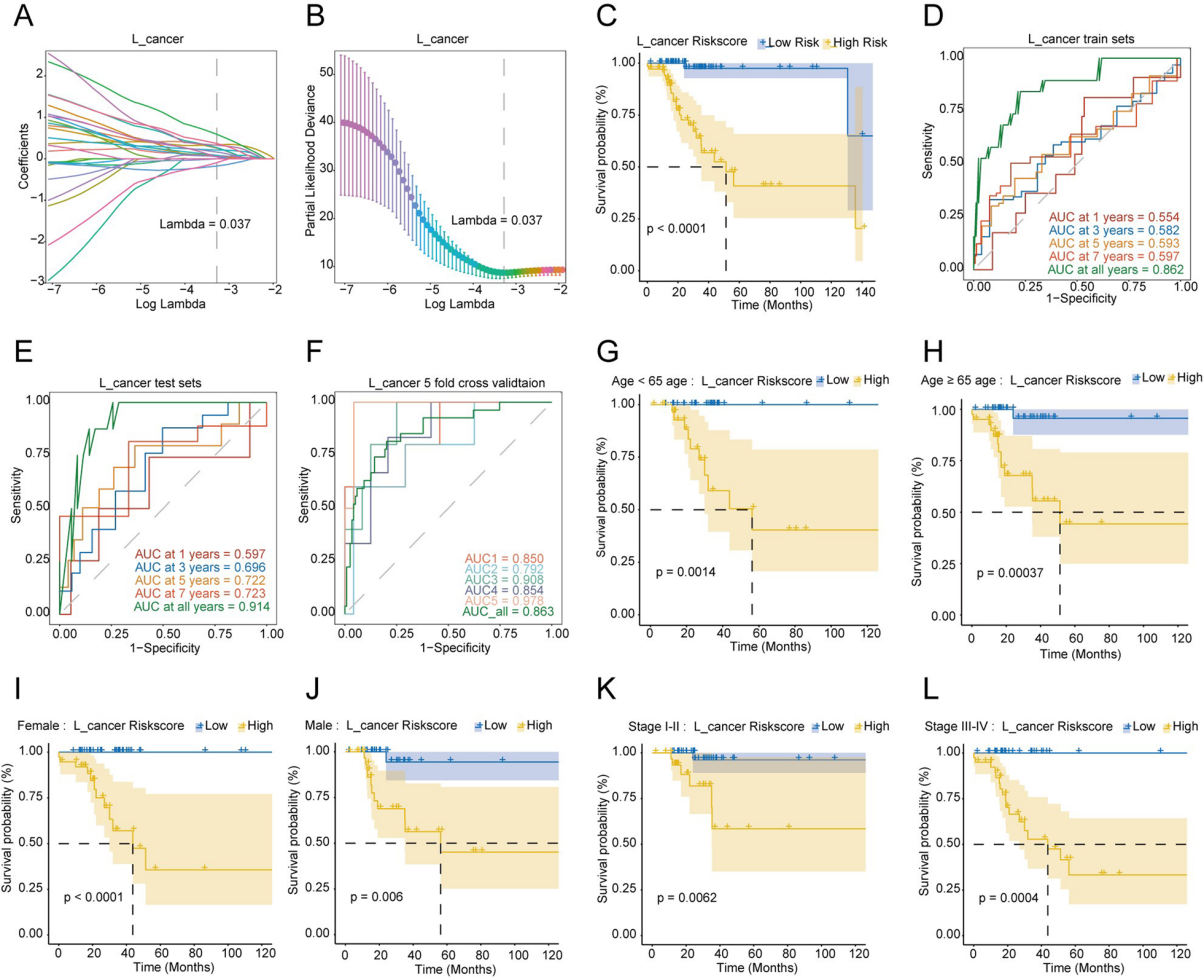
Construction and validation of the prognostic model in L_cancer group. **A** LASSO coefficient profiles of DEGs. **B** Selection of the optimal parameter (lambda) in the LASSO model. **C** Differences in overall survival between high-risk and low-risk groups based on the risk scores in L_cancer patients. **D** Time-dependent ROC curves in the training set at 1-year, 2-year, 3-year, 5-year, 7-year and all-year in L_cancer patients. **E** Time-dependent ROC curves in the testing set at 1-year, 2-year, 3-year, 5-year, 7-year and all-year in L_cancer patients. **F** The ROC curves of Five-fold cross-validation in L_cancer patients. **G**–**L** Comparison of survival rates of high-risk and low-risk groups in different clinical subtypes in L_cancer patients. Survival analysis of different clinical characteristics including **G** Age < 65, **H** Age ≥ 65, **I** Female, **J** Male, **K** Stage I-II, **L** Stage III-IV

The L_cancer patient prognosis features and risk score were calculated as: KNG1 × 0.621 + CYP11A1 × 0.600 + SMPD1 × 1.370 + DAND5 × 0.859 + NKPD1 × 0.721 + RP11-59D5_B.2 × 0.568 + CTD-2184C24.2 × 0.514 + RP11-680F8.3 × 0.517 − RP11-51F16.9 × 0.731 + CTD-2012K14.8 × 0.765 (Fig. 6A, B). The cutoff of risk score is 7.801, which had a great impact on OS (Fig. 6C). Scores lower than 7.801 have been defined as low-risk L_cancer patients, while scores higher than 7.801 have been defined as high-risk L_cancer patients. The AUC values of the risk score in the training set for 1-year, 3-year, 5-year, 7-year, and all-time OS were 0.554, 0.582, 0.593, 0.597, and 0.862, respectively (Fig. 6D).

**Fig. 7:**
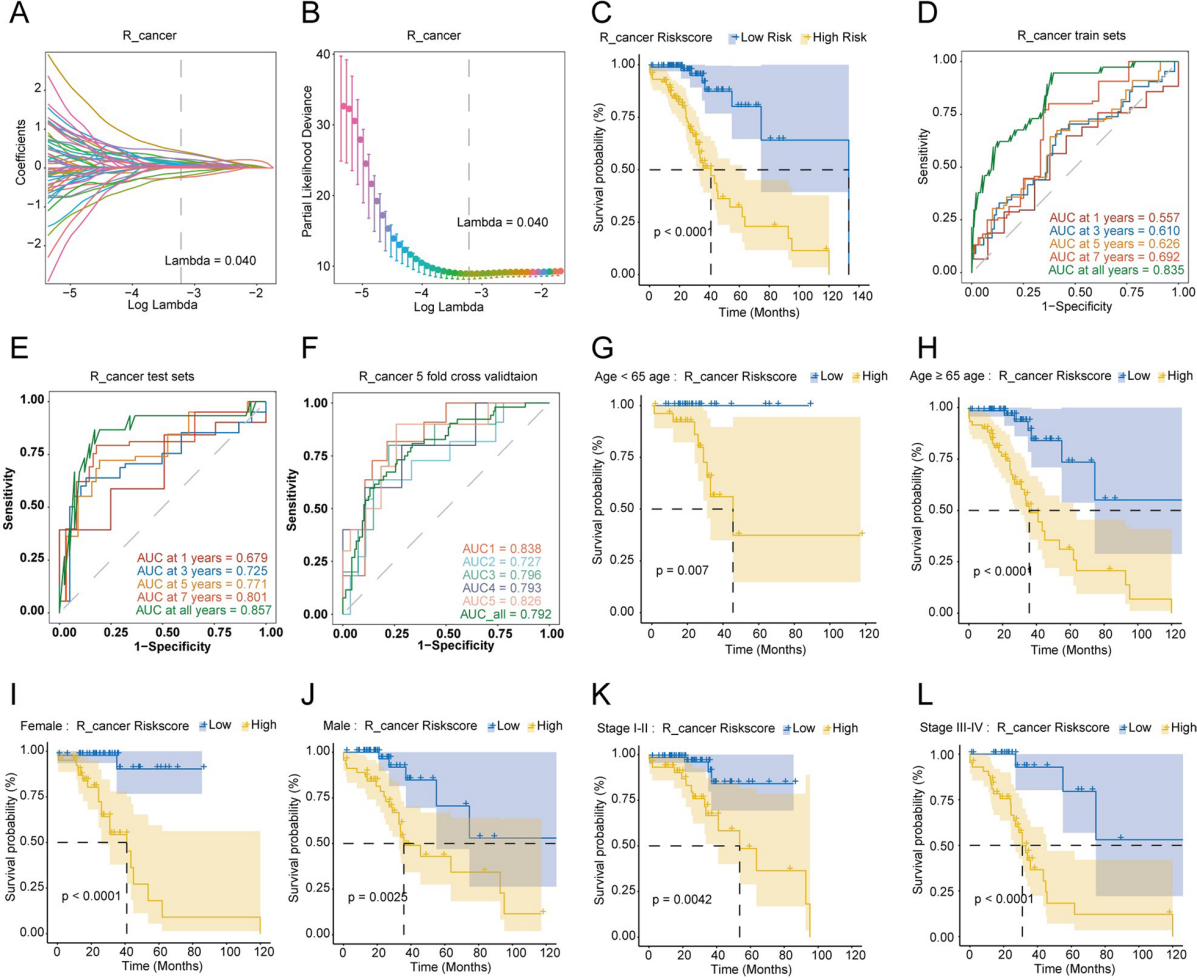
Construction and validation of the prognostic model in R_cancer group. **A** LASSO coefficient profiles of DEGs. **B** Selection of the optimal parameter (lambda) in the LASSO model. **C** Differences in overall survival between high-risk and low-risk groups based on the risk scores in R_cancer patients. **D** Time-dependent ROC curves in the train set at 1-year, 2-year, 3-year, 5-year, 7-year, and all-year in R_cancer patients. **E** Time-dependent ROC curves in the test set at 1-year, 2-year, 3-year, 5-year, 7-year, and all-year in R_cancer patients. **F** The ROC curves of Five-fold cross-validation in R_cancer patients. **G**–**L** Comparison of survival rates of high-risk and low-risk groups in different clinical subtypes in R_cancer patients. Survival analysis of different clinical characteristics including **G** Age < 65, **H** Age ≥ 65, **I** Female, **J** Male, **K** Stage I-II, **L** Stage III-IV

The R_cancer prognosis features and risk score were calculated as: MOCS1 × 1.100 − PTGS2 × 0.722 + PLEKHA8P1 × 0.409 − ZC3H12C × 0.571 + LPO × 0.575 + METTL11B × 0.294 + RP11-278A23.1 × 0.508 + RP11-452K12.7 × 0.405 − RP11-742B18.1 × 0.360 + RP11-626H12.2 × 0.787 (Fig. 7A, B). The cutoff of risk score is 11.981, which had a great impact on OS (Fig. 7C). Scores lower than 1.981 have been defined as low-risk R_cancer patients, while scores higher than 1.981 have been defined as high-risk R_cancer patients. The AUC values of the risk score in the training set for 1-year, 3-year, 5-year, 7-year, and all-time OS were 0.557, 0.610, 0.626, 0.692, and 0.835, respectively (Fig. 7D).

### Internal validation of the prognosis genes model and stratified analysis by clinical factors

The efficacy of the prognostic signature was validated using a testing set of TCGA patients. Five-fold cross-validation was used to assess the stability of the model.

Among the L_cancer patients, the area under the curve (AUC) values of risk scores predicted in the testing set for 1-year, 3-year, 5-year, 7-year, and all-time OS were 0.597, 0.696, 0.722, 0.723, and 0.914, respectively (Fig. 6E). The AUC values of fivefold cross-validation were 0.860, 0.792, 0.908, 0.854, and 0.978, respectively, and the integrated AUC value was 0.863 (Fig. 6F). The results revealed that the AUC values of fivefold cross-validation were high and similar, indicating that the model had good predictability and stability. Based on the obtained sample clinical characteristics, patients were stratified into age < 65 years and age ≥ 65 years sub-groups (Fig. 6G, H), female and male sub-groups (Fig. 6I, J), and pathological tumor Stage I/II and Stage III/IV sub-groups (Fig. 6K, L). The overall survival analysis was performed in each sub-group, based on the level of risk score, and all results showed statistical differences.

Likewise, in R_cancer patients, the AUC values of risk scores predicted in the test set for 1-year, 3-year, 5-year, 7-year, and all-time OS were 0.679, 0.725, 0.771, 0.801, and 0.857, respectively (Fig. 7E). The AUC values of fivefold cross-validation were 0.838, 0.727, 0.796, 0.793, and 0.826, respectively, and the integrated AUC value was 0.792 (Fig. 7F). The results revealed that the AUC values of fivefold cross-validation were high and similar, indicating the model had good predictability and stability. Patients were also stratified into age < 65 years and age ≥ 65 years sub-groups (Fig. 7G, H), female and male sub-groups (Fig. 7I, J), and pathological tumor Stage I/II and Stage III/IV sub-groups (Fig. 7K, L). Overall survival analysis was also performed in each sub-group, based on the level of risk score, and all the results showed statistical differences.

### Incorporating clinical factors to develop individualized nomograms

**Fig. 8:**
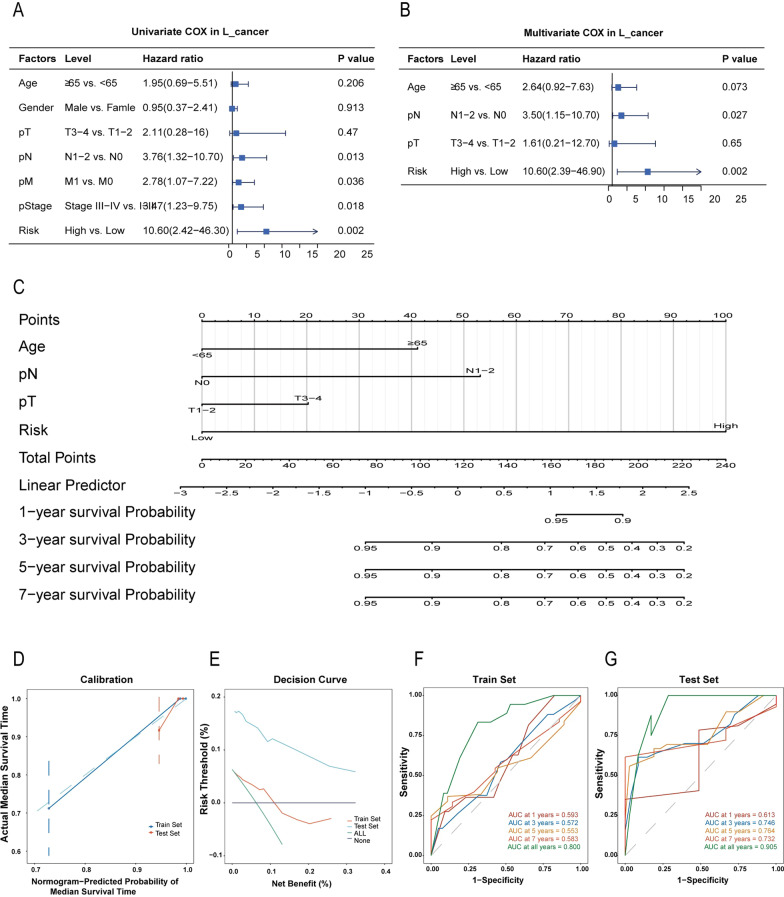
Validation of the nomogram in predicting the overall survival of L_cancer in the TCGA dataset. **A**, **B** Univariate and multivariate Cox regression analysis of L_cancer prognostic signatures and clinical characteristics. **C** Developed incorporating clinical factors nomogram of L_cancer patients. **D** Calibration curve of the nomogram in the train set and test set of L_cancer patients. **E** Decision curve analysis of the nomogram in the train set and test set of L_cancer patients. **F** Time-dependent ROC curves in the train set at 1-year, 2-year, 3-year, 5-year, 7-year, and all-year in L_cancer patients. **G** Time-dependent ROC curves in the test set at 1-year, 2-year, 3-year, 5-year, 7-year, and all-year in L_cancer patients

**Fig. 9:**
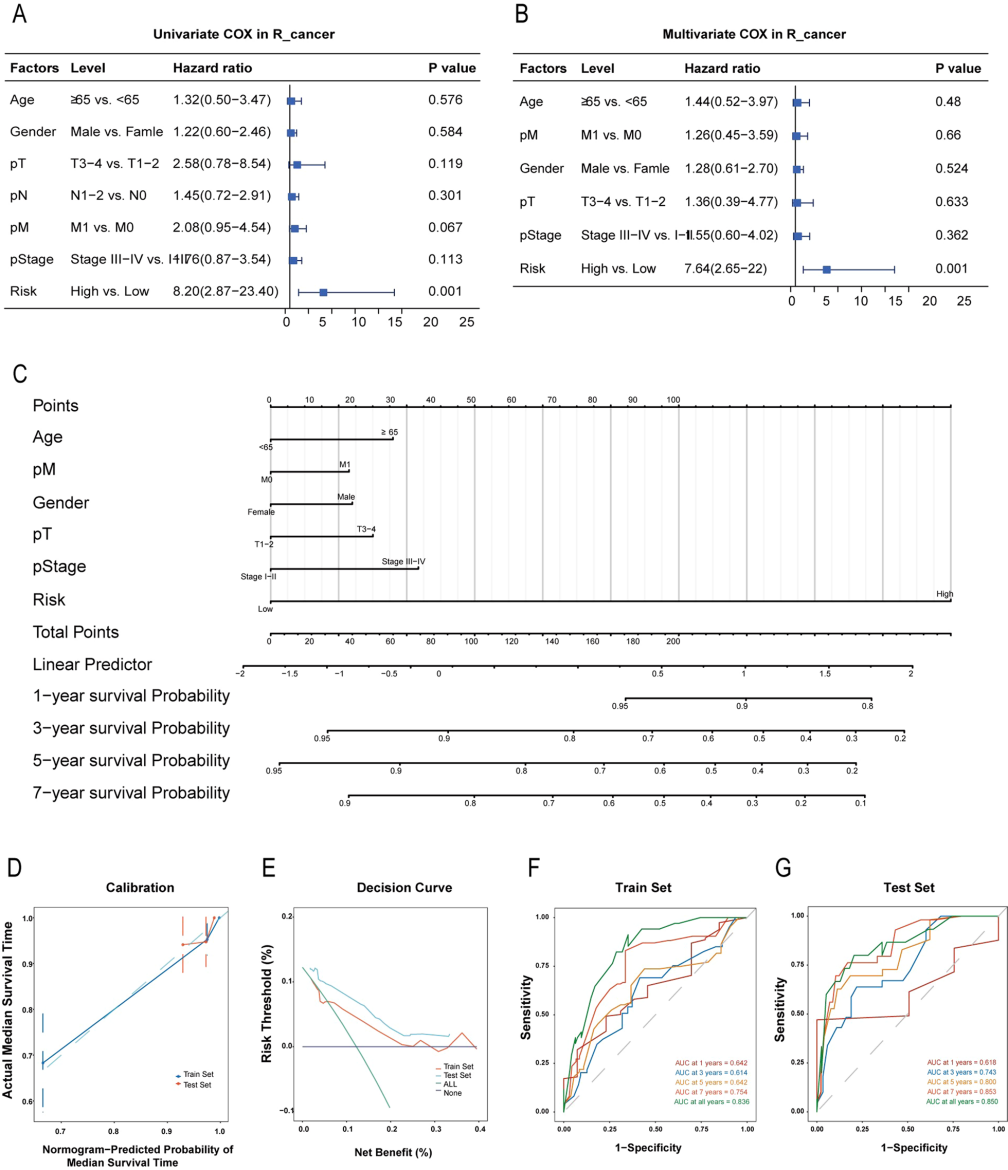
Validation of the nomogram in predicting overall survival of R_cancer in the TCGA dataset. **A, B** Univariate and multivariate Cox regression analysis of R_cancer prognostic signatures and clinical characteristics. **C** Developed incorporating clinical factors nomogram of R_cancer patients. **D** Calibration curve of the nomogram in the train set and test set of R_cancer patients. **E** Decision curve analysis of the nomogram in the train set and test set of R_cancer patients. **F** Time-dependent ROC curves in the train set at 1-year, 2-year, 3-year, 5-year, 7-year, and all-year in R_cancer patients. **G** Time-dependent ROC curves in the test set at 1-year, 2-year, 3-year, 5-year, 7-year, and all-year in R_cancer patients

Clinical characteristics, including Age, Gender, T, N, M, Stage, and risk score, were utilized to perform univariate analyses in the training sets of L_cancer (Fig. 8A) and R_cancer (Fig. 9A), respectively. After statistical adjustment for other variables with multivariate Cox regression analysis, we found that the Risk, pathological M, pathological stage, gender, and age were the only six independent prognostic factors that could be used to predict the survival rate in L_cancer (Fig. 8B), while the Risk, pathological N, pathological T, and age were the only four independent prognostic factors that could be used to predict the survival rate in R_cancer. (Fig. 9B). L_cancer patients’ nomogram (Fig. 8C) and R_cancer patients’ nomogram (Fig. 9C) were developed using the above prognostic features, with the total points calculated by adding the points of individual prognostic features.

### Predictive performance of the established nomogram

Among L_cancer patients, the calibration curve and decision curve analysis for predicting median survival time OS in the training and testing sets indicated that the nomogram-predicted survival similarly corresponded with actual survival outcomes (Fig. 8D, E). The AUC of the nomogram was 0.8 in the training set and 0.905 in the testing set (Fig. 8F, G).

In R_cancer patients, the calibration curve and decision curve analysis for predicting median survival time OS in the training and testing sets indicated that the nomogram-predicted survival similarly corresponded with actual survival outcomes (Fig. 9D, E). The AUC of the nomogram was 0.836 in the training set and 0.850 in the testing set. (Fig. 9F, G).

### External validation of the prognosis signature by qRT-PCR

**Fig. 10:**
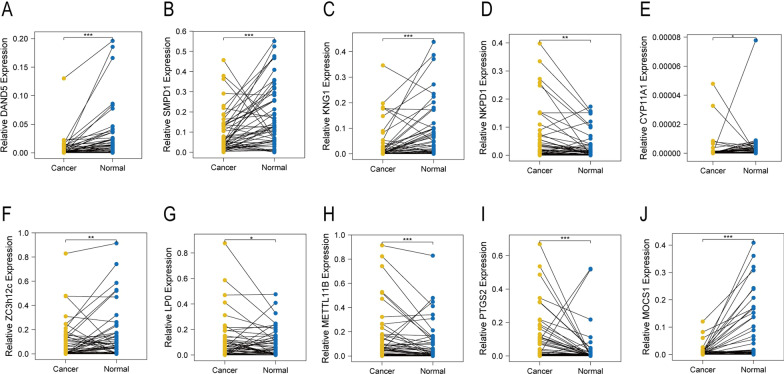
**A**–**E** qRT-PCR validation of the expression of DAND5, SMPD1, KNG1, NKPD1, and CYP11A1 in 50 pairs of L_cancer patients. **F**–**J** qRT-PCR validation of the expression of ZC3h12c, LPO, METTL11B, PTGS2, and MOCS1 in 50 pairs of R_cancer patients. *Notes*: *P < 0.05, **P < 0.01, ***P < 0.001

The obtained results were further validated by qRT-PCR, as shown in Fig. 10.

In 51 pairs of L_cancer patients, compared with adjacent cancer tissues, the expression of DAND5, SMPD1, KNG1, NKPD1, and CYP11A1 were found to be down-regulated in cancer tissues (two-tailed paired t-test; all *P* < 0.05, Fig. 10A–E).

Moreover, in 51 pairs of R_cancer patients, compared with adjacent cancer tissues, the expression of LPO, METTL11B, and PTGS2 were found to be up-regulated, and ZC3H12C and MOCS1 were down-regulated in cancer tissues (two-tailed paired t-test; all *P* < 0.05, Fig. 10F–J).

### Differences in the immune microenvironment, TMB landscape, immune checkpoint-related genes, and HLA-family genes level between high- and low-risk patients

Based on the difference in the immune microenvironment and TMB landscape between left and right CRC, we next analyzed the difference in these aspects between high- and low-risk patients based on prognostic gene models.

**Fig. 11:**
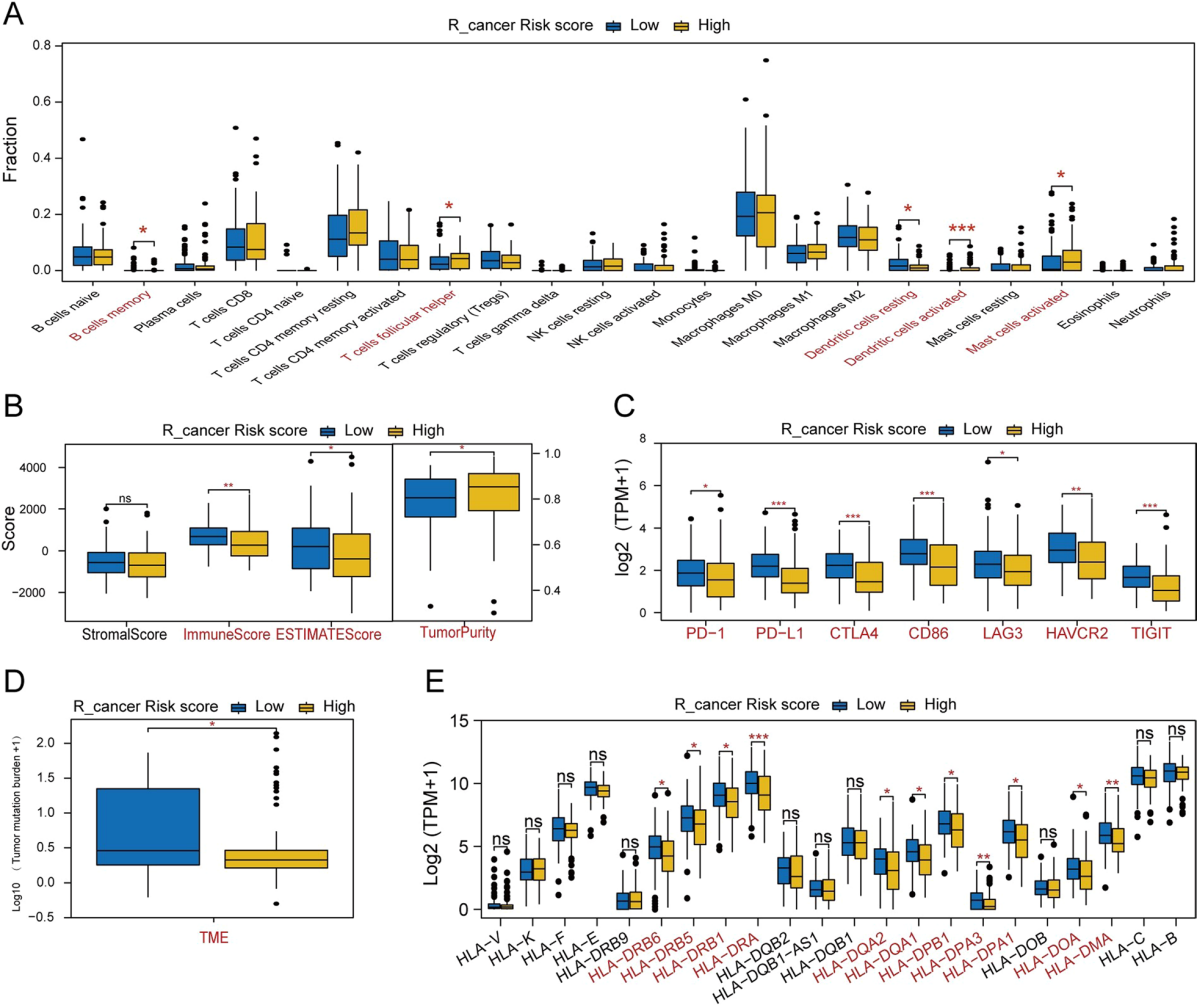
**A** The comparison of immune infiltration levels between high-risk and low-risk groups in R_cancer patients, based on CIBERSORT. **B** The Stromal Score difference, Immune Score difference, ESTIMATE Score difference, and tumor purity difference between high-risk and low-risk groups in R_cancer patients. **C** The immune checkpoint-related gene expression levels in high-risk and low-risk groups in R_cancer patients. **D** The tumor mutation burden difference between high-risk and low-risk groups in R_cancer patients. **E** HLA-related gene expression level between high-risk and low-risk groups in R_cancer patients. *Notes*: ns *P* > 0.05, * *P* < 0.05, ** *P* < 0.01, *** *P* < 0.001

In the R_cancer patients, high-risk patients had a lower proportion of ‘B cells memory’, ‘Dendritic cells resting’, immune score, ESTIMATE score, immune checkpoint-related genes, and HLA-family genes, and a higher proportion of ‘T cells follicular helper’, ‘Dendritic cells activated’, and ‘Mast cells activated’ (Wilcoxon test, *P* < 0.05; Fig. 11A–E). These results indicate that R_cancer patients in high- and low-risk groups may have different responses to immunotherapy, and immunotherapy in R_cancer low-risk patients may be more beneficial.

In the L_ancer patients, there was no difference in these indicators between high- and low-risk patients (Additional file 2: Fig. S2A–E).

### Correlation of hub gene and risk score with immune-related score and genes

**Fig. 12:**
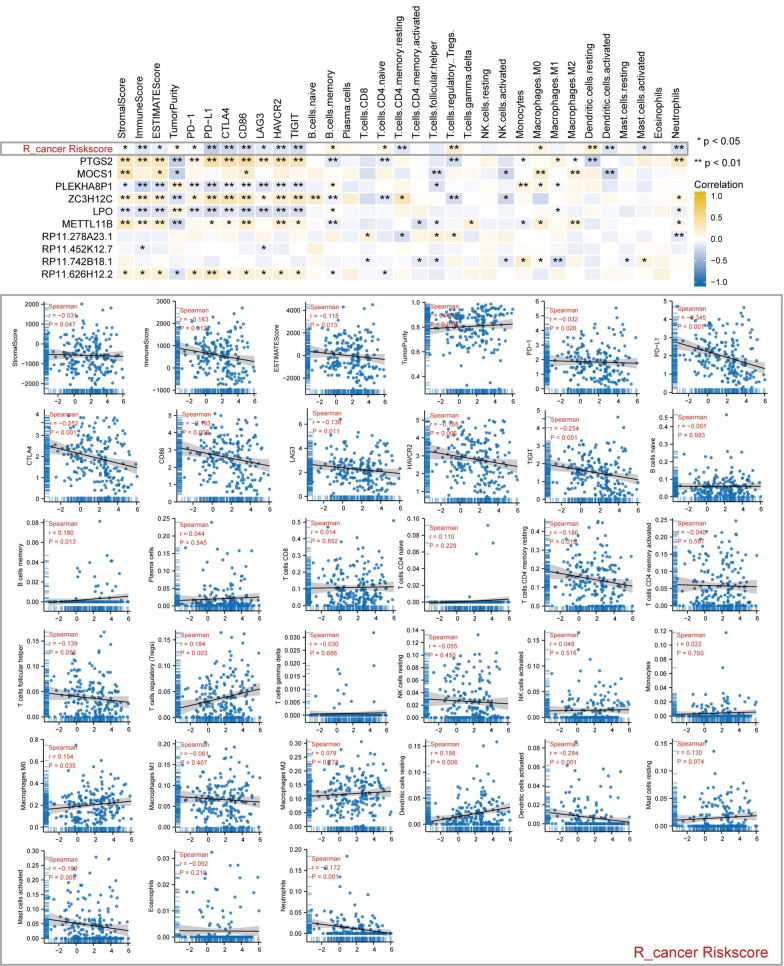
Show the correlation of R_cancer RiskScore and R_cancer hub genes expression with immune infiltration level in R_cancer patients

Correlation analyses were carried out for risk scores and hub genes with immune-related scores and genes. As we can see, in R_cancer patients, R_cancer risk score was strongly correlated with immune-related scores and genes (Fig. 12). In particular, it has a significant negative correlation with immune checkpoint-related genes, Stromal score, immune score, and ESTIMATE score and a positive correlation with tumor purity. These results prove that R_cancer patients with R_cancer low-risk score may benefit more from immunotherapy. In addition, the R_cancer risk score was positively associated with the content of ‘B cells memory’, ‘T cells CD4 naïve’, ‘T cells regulatory Tregs’, ‘Macrophages M0’, and ‘Dendritic cells resting’ and negatively associated with the content of ‘T cells follicular helper’, ‘Dendritic cells activated’, ‘Mast cells activated’ and ‘Neutrophils’. In L_cancer patients, L_cancer risk score was no correlation with immune-related scores and genes (Additional file 3: Fig. S3).

## Discussion

CRC has a heterogeneous tumor composition and complex oncogenic mechanisms. The development of individualized treatment strategies and the evaluation of patient prognoses based on tumor location are crucial. This study is the first to separately build predictive models for L_cancer and R_cancer, to the best of our knowledge. We presented two nomograms for CRC classified with respect to both tumor side and location based on prognostic gene signatures and clinical prognostic factors can be used to distinguish high-risk from low-risk patients effectively. The L_cancer nomogram includes prognostic genes (KNG1, CYP11A1, SMPD1, DAND5, NKPD1, RP11-59D5_B.2, CTD-2184C24.2, RP11-680F8.3, RP11-51F16.9, CTD-2012K14.8), pathological N, pathological T, and age, which can be used to predict the survival rate; meanwhile, the R_cancer nomogram comprises prognostic genes (MOCS1, PTGS2, PLEKHA8P1, ZC3H12C, LPO, METTL11B, RP11-278A23.1, RP11-452K12.7, RP11-742B18.1, RP11-626H12.2), age, pathological M, pathological T, pathological stage, and gender, which can also be used to predict the survival rate.

Numerous studies have confirmed that the right- and left-sided colons are distinct due to their embryological origins. The right-side colon originate from the midgut, whereas the left-side colon originate from the hindgut. In this study, we confirmed that there exist significant differences in the TMB and immune microenvironment between right- and left-sided CRC patients. Furthermore, right-sided CRC tend to have worse prognosis than left-sided CRC patients. The difference between right- and left-sided CRC patients' survival rates is might be caused by the higher frequency of mutations in addition to changes in the tumor microenvironment associated with tumor purity. According to recent research, mutation prevalence differs depending on side and location. RAS mutations declined from 70% in patients with right-sided CRC to 43% in those with left-sided CRC, while the number of BRAFV600 mutations increased from 10 to 22% between the same locations. Sigmoid and rectal tumors with left-sided mutations were more likely to harbor TP53 mutations than PIK3CA, BRAF, or CTNNB1 mutations [3]. Consistent with our results, in left-sided tumors, TP53 (L_cancer: 68%, R_cancer: 48%) showed a higher mutation rate; meanwhile, in right-sided tumors, PIK3CA (L_cancer: 18%, R_cancer: 33%) and KRAS (L_cancer: 36%, R_cancer: 46%) showed higher yield mutation rates. The results in our study align well with a recent report by Marshall et.al., who also demonstrated significant differences between L_cancer and R_cancer in mutation patterns.

The tumor microenvironment (TME) refers to the physical environment around a tumor, including the immune cells, neurons, blood vessels, extracellular matrix, and other cellular functions related to tumor progression and therapy effects. We also confirmed that the immune microenvironment affects the prognosis of patients with CRC. Aggressively growing tumors create a highly immunosuppressive TME that depletes antitumor responses and promotes tumor progression [19, 20].

Based on the Estimation of STromal and Immune cells in MAlignant Tumor tissues using Expression data approach, immune score and tumor purity can reveal information about the tumor's immune status. Low immune scores and high tumor purity have been associated with better prognoses in several studies [21–23]. Based on this, we examined the differences in tumor immune microenvironment between right- and left-sided CRC patients. In our study, L_cancer patients not only had poor prognosis but also had high ESTIMATE and immune scores, as well as low tumor purity. Thus, we further analyzed the effect of high- or low-risk on immune infiltration in patients in both L_cancer and R_cancer models. We found that, in the R_cancer model, high-risk patients had lower immune and ESTIMATE scores and higher tumor purity than low-risk patients. However, there was no difference between high- and low-risk in the L_cancer model with respect to immune infiltration. Besides, in the R_cancer model, high-risk patients were significantly different from low-risk patients in terms of immune infiltrating cell types, such as memory B-cells, dendritic, T follicular helper cells and mast cell activation. Nevertheless, in the L_cancer model, the high- and low-risk patients showed no difference. These results may be related to our different models for L_cancer and R_cancer. The findings of some studies were in line with our study, where low tumor purity result in poor prognosis in glioma and CRC [21, 22]. Additionally, the proportions of CD8 T-cells and T follicular helper cells were significantly higher in the R_cancer group, while M0 macrophages had higher infiltration in L_cancer groups. A recent single-cell RNA-Seq study between right- and left-sided CRC patients discussed the difference in single-cell transcriptomes between the two groups, which was in line with our findings. In summary, there has been increasing awareness of the body's ability to fight tumors through various types of cells cytokines, and chemokines. Immune cells, especially, play a critical role in this. Immunotherapy has become increasingly popular as a treatment option for cancer patients with refractory malignant tumors, which can benefit significantly from immune checkpoint inhibitors. To determine whether immunotherapy is effective, TMB, TME, and immune checkpoint levels are considered as biomarkers [23–25]. A previous study has demonstrated that, in CRC patients, the prognostic impact of PD-L1 and PD-1 expression varies according to the primary tumor site. Moreover, the presence level of PD-L1 is an independent prognostic factor for right-side tumors [26]. This finding was in line with our study, which demonstrated that there were significant differences in PD-1, PD-L1, and CTLA4 expression between right- and left-sided CRC patients.

Given this, this study independently assessed the effect of the tumor microenvironment in L_cancer and R_cancer of high- and low-risk patients from two aspects (TMB and immune microenvironment), leading us to speculate that R_cancer—especially low-risk R_cancer—patients may benefit more from immunotherapy [27, 28]. Validation is needed, but these results could be clinically significant as they indicate that tumor location is important to consider in therapeutic decisions, including eligibility for immunotherapy.

The hub genes in the signature have previously been shown to be potential biomarkers. Relevant research has reported that PTGS2-driven inflammatory responses can induce tumor expression of microRNA-21, which can increase the level of the inflammatory mediator prostaglandin E2 (PGE2) by down-regulating PGE2-metabolizing enzymes, contributing to colorectal cancer development [28–32]. PLEKHA8P1 expression has been associated with the development and progression of many malignancies in humans, such as CRC and renal cancer [33]; moreover, research has shown that its dysregulated expression affects 5-Fluorouracil-induced chemoresistance in the human hepatocellular carcinoma cell line FT3-7 [34]. Prior studies found ZC3H12A has links with immune homeostasis and post-transcriptional regulation which can stimulate tumor progression in lung and colon cancer [35–37]. LPO can collaborate with activated Wnt signaling to induce intestinal neoplasia [38]. METTL11B expression has been associated with poor prognosis in colorectal cancer and is higher in cancer tissues than in neighboring normal tissues [39]. NKPD1 has been predicted to be linked with the de novo synthesis of sphingolipids [40]. Increased DAND5 level is an independent risk factor for both colorectal and breast cancers and the prediction of poor prognoses [41, 42]. SMPD1 encodes lysosomal acid sphingomyelinase, which converts sphingomyelin to ceramide. Prior studies have found that the functional inhibition of acid sphingomyelinase contributes to tumor cell death by overactivation of hypoxia stress-response pathways [43]. Another study has shown that down-regulation of SMPD1 is linked with resistance to chemotherapy regimens including 5-Fluorouracil [44]. Studies have shown CYP11A1, which can hydroxylate the side-chain of vitamin D3 at carbons 17, 20, 22, and 23, are related to susceptibility to breast cancer [45, 46]. KNG1 can regulate the expressions of VEGF, cyclinD1, ki67, and caspase-3/9, exerting anti-angiogenic properties and inhibiting the proliferation of endothelial cells. Over-expression of KNG1 can inhibit the activity of PI3K/Akt, decrease tumor growth, and promote apoptosis [47]. On the contrary, other researchers have found that KNG1 expression was significantly increased in colorectal cancer lesions [48]. At present, there has been no reported association between MOSC1, RP11-278A23.1, RP11-452K12.7, RP11-742B18.1, RP11-626H12.2 RP11-59D5_B.2, CTD-2184C24.2, RP11-680F8.3, RP11-51F16.9, CTD-2012K14.8, and cancer. In the end, RT-qPCR was performed to verify the results from the bioinformatic analyses of LCC and RCC. We revealed that the prognostic gene expression results were consistent with the outcomes of our survival analysis, indicating that our results are reproducible and reliable. In addition, this further confirmed that these key genes are related to the occurrence and development of colon cancer.

This study had some limitations. The signatures and nomograms constructed in this study using vast datasets from TCGA and our patient database were robust, but the study was still a retrospective one. Second, we explored the TMB and immune microenvironment landscape between right- and left-sided CRC patients and between patients in different risk groups; however, the study lacked experimental verification. Third, as previously noted, obtaining risk scores requires knowledge of ten genes expressed in tumor tissues, thereby increasing the difficulty of applying the nomograms. It appears that many molecular diagnostic or prognostic models have the same problem. Researchers and clinicians need to figure out how to simplify the application of these models in clinical settings. In the future, molecular detection technology may solve this dilemma. The constructed nomograms may be used routinely.

## Conclusions

We found significant differences between L_cancer and R_cancer patients, including clinical features, transcriptome, TMB, immune microenvironment landscape, suggesting that colon cancer can be classified and analyzed into different clinical types with respect to their differences in anatomical location and gene expression, thus aiding in the early diagnosis and prognosis of colon cancer. We established two clinical predictive nomograms in combination with clinical features to provide a basis for the personalized and precise treatment of L_cancer and R_cancer. These hub genes may become promising biomarkers for the diagnosis, treatment, and prognosis of colon cancer. Moreover, The findings support previous studies suggesting that proximal and distal CRC can be classified differently in terms of epidemiology, pathology, and genetics.

## Supplementary Information

**Additional file 1: Fig. S1.** (A) Kaplan-Meier survival analysis of ten hub genes (KNG1, CYP11A1, SMPD1, DAND5, NKPD1, RP11-59D5_B.2, CTD-2184C24.2, RP11-680F8.3, RP11-51F16.9, CTD-2012K14.8) in L_cancer patients between high-expression and low-expression groups. (B) Kaplan-Meier survival analysis of ten hub genes (MOCS1, PTGS2, PLEKHA8P1, ZC3H12C, LPO, METTL11B, RP11-278A23.1, RP11-452K12.7, RP11-742B18.1, RP11-626H12.2) in R_cancer patients between high-expression and low-expression groups.

**Additional file 2: Fig. S2.** (A) The comparison of immune infiltration levels between high-risk and low-risk groups in L_cancer patients, based on CIBERSORT. (B) The Stromal Score difference, Immune Score difference, ESTIMATE Score difference, and tumor purity difference between high-risk and low-risk groups in L_cancer patients. (C) The immune checkpoint-related gene expression levels in high-risk and low-risk groups in L_cancer patients. (D) The tumor mutation burden difference between high-risk and low-risk groups in L_cancer patients. (E) HLA-related gene expression level between high-risk and low-risk groups in L_cancer patients. (Notes: ns P>0.05).

**Additional file 3: Fig. S3.** Show the correlation of L_cancer RiskScore and L_cancer hub genes expression with immune infiltration level in L_cancer patients.

## Data Availability

The datasets used and/or analyzed during the current study are available from TCGA repository: https://portalgdccancer.gov.
