# Supplementary figures and images for: Comprehensive analysis of the differences between left- and right-side colorectal cancer and respective prognostic prediction
Source: BMC Gastroenterol. 2022 Nov 23;22:482. doi: 10.1186/s12876-022-02585-3 (PMC9685975; doi:10.1186/s12876-022-02585-3)

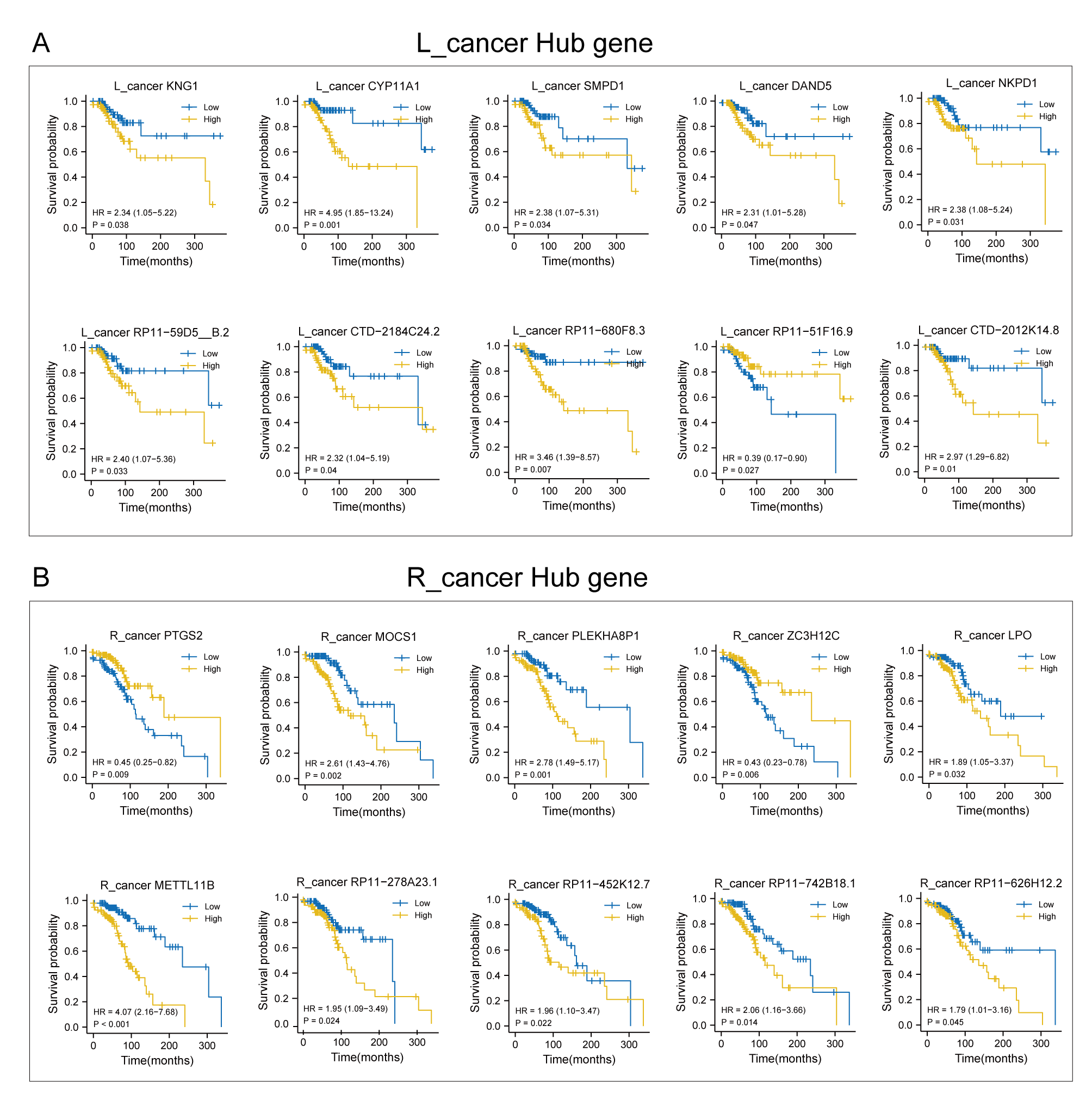

Supplement: Supplementary file 1 — Additional file 1: Fig. S1. (A) Kaplan-Meier survival analysis of ten hub genes (KNG1, CYP11A1, SMPD1, DAND5, NKPD1, RP11-59D5_B.2, CTD-2184C24.2, RP11-680F8.3, RP11-51F16.9, CTD-2012K14.8) in L_cancer patients between high-expression and low-expression groups. (B) Kaplan-Meier survival analysis of ten hub genes (MOCS1, PTGS2, PLEKHA8P1, ZC3H12C, LPO, METTL11B, RP11-278A23.1, RP11-452K12.7, RP11-742B18.1, RP11-626H12.2) in R_cancer patients between high-expression and low-expression groups. [file 12876_2022_2585_MOESM1_ESM.tif]

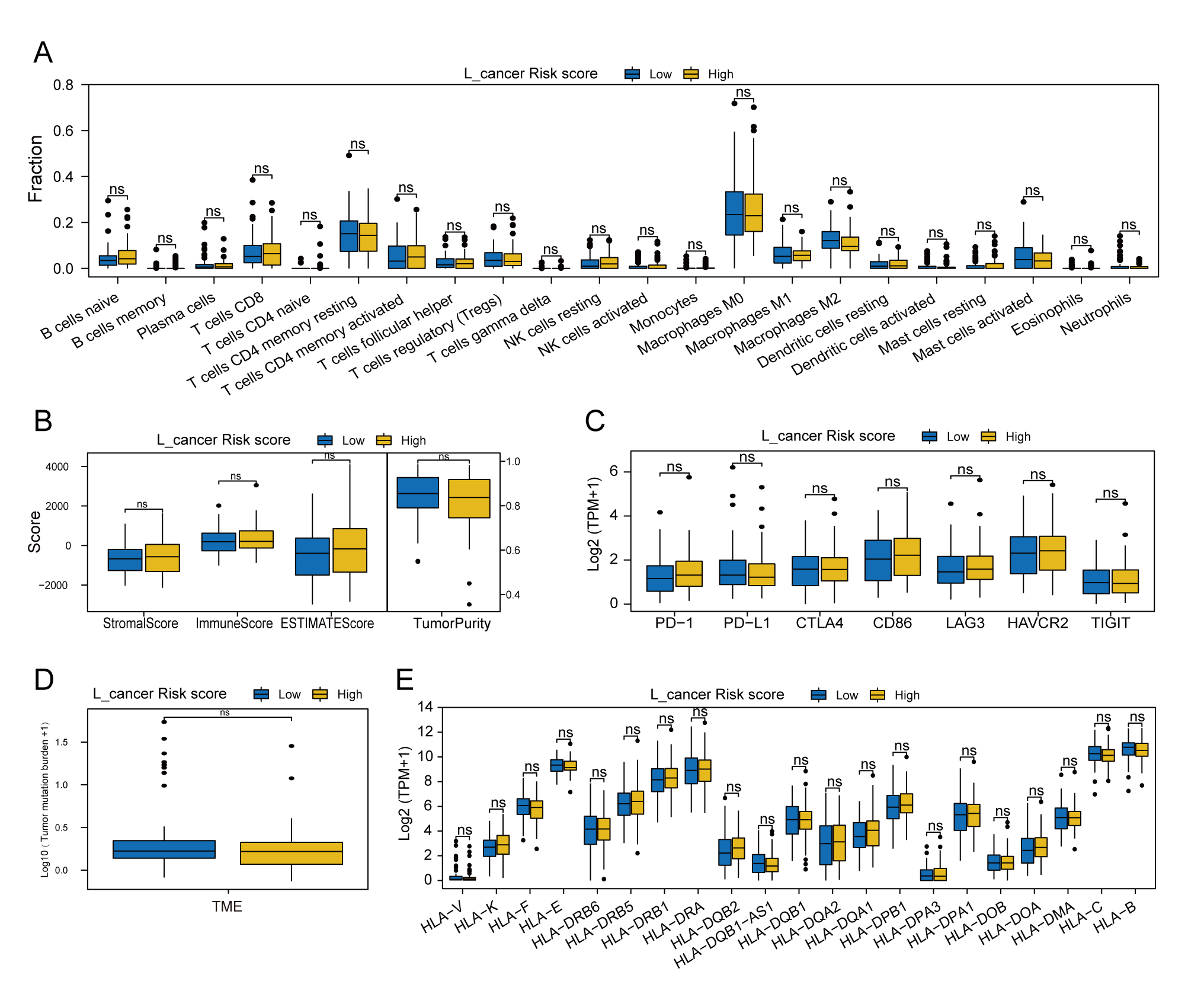

Supplement: Supplementary file 2 — Additional file 2: Fig. S2. (A) The comparison of immune infiltration levels between high-risk and low-risk groups in L_cancer patients, based on CIBERSORT. (B) The Stromal Score difference, Immune Score difference, ESTIMATE Score difference, and tumor purity difference between high-risk and low-risk groups in L_cancer patients. (C) The immune checkpoint-related gene expression levels in high-risk and low-risk groups in L_cancer patients. (D) The tumor mutation burden difference between high-risk and low-risk groups in L_cancer patients. (E) HLA-related gene expression level between high-risk and low-risk groups in L_cancer patients. (Notes: ns P>0.05). [file 12876_2022_2585_MOESM2_ESM.tif]

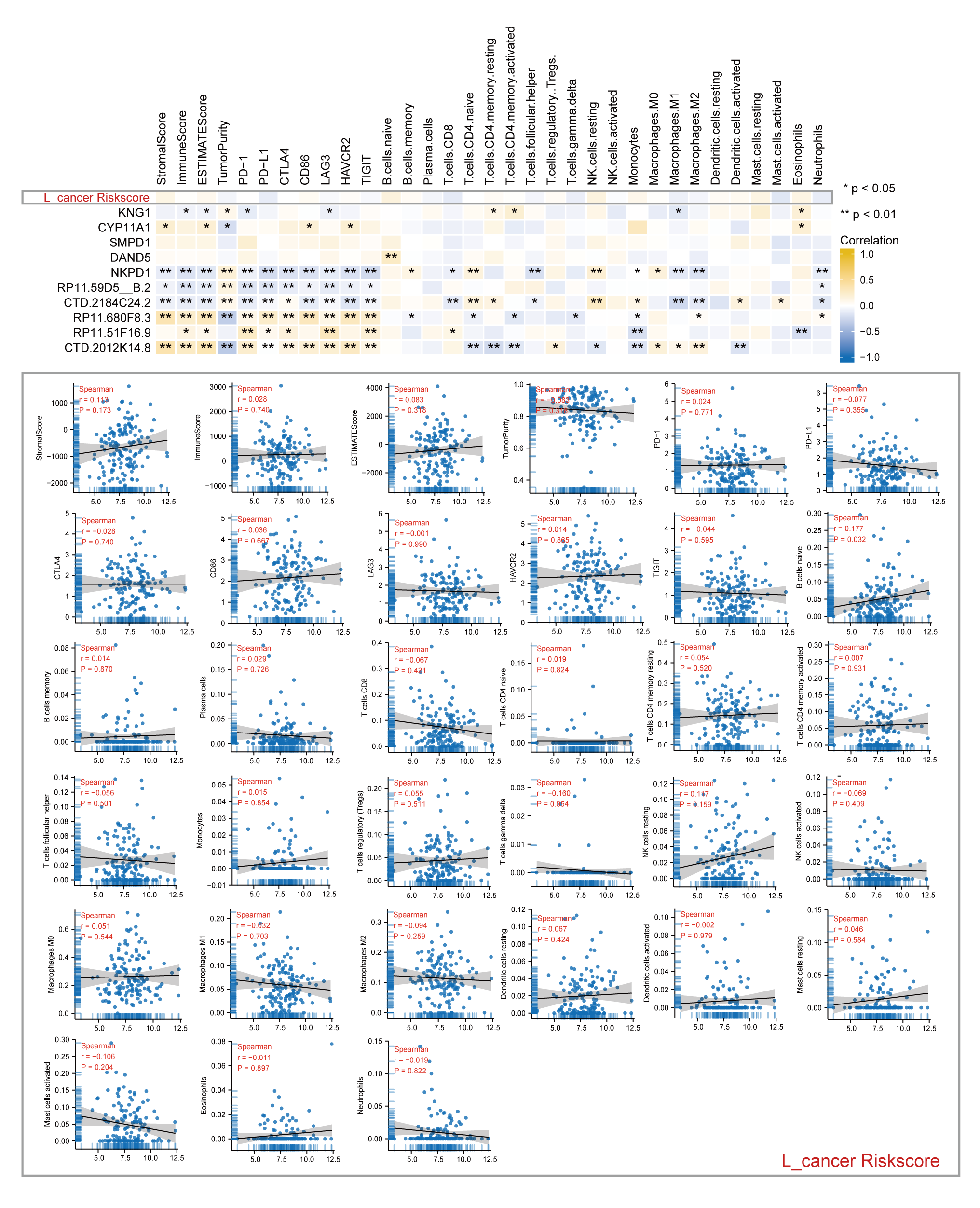

Supplement: Supplementary file 3 — Additional file 3: Fig. S3. Show the correlation of L_cancer RiskScore and L_cancer hub genes expression with immune infiltration level in L_cancer patients. [file 12876_2022_2585_MOESM3_ESM.tif]
